# Identification of intratumoral bacteria that enhance breast tumor metastasis

**DOI:** 10.1128/mbio.03595-24

**Published:** 2025-02-11

**Authors:** Zachary J. Gerbec, Antonio Serapio-Palacios, Avril Metcalfe-Roach, Zakhar Krekhno, Haggai Bar-Yoseph, Sarah E. Woodward, Jorge Pena-Díaz, Oksana Nemirovsky, Shannon Awrey, Sebastian H. Moreno, Sean Beatty, Esther Kong, Nina Radisavljevic, Mihai Cirstea, Shawn Chafe, Paul C. McDonald, Sam Aparicio, B. Brett Finlay, Shoukat Dedhar

**Affiliations:** 1 Department of Integrative Oncology, BC Cancer Research Institute, Vancouver, British Columbia, Canada; 2 Michael Smith Laboratories, University of British Columbia, Vancouver, British Columbia, Canada; 3 Department of Microbiology and Immunology, University of British Columbia, Vancouver, British Columbia, Canada; 4 Department of Biochemistry and Molecular Biology, University of British Columbia, Vancouver, British Columbia, Canada; 5 Department of Molecular Oncology, BC Cancer Research Institute, Vancouver, British Columbia, Canada; 6 Michael Smith Genome Science Centre, BC Cancer Research Institute, Vancouver, British Columbia, Canada; Columbia University, New York, New York, USA

**Keywords:** tumor microbiome, metastasis, cancer, intratumoral bacteria, *Bacillus*

## Abstract

The central, mortality-associated hallmark of cancer is the process of metastasis. It is increasingly recognized that bacteria influence multiple facets of cancer progression, but the extent to which tumor microenvironment-associated bacteria control metastasis in cancer is poorly understood. To identify tumor-associated bacteria and their role in metastasis, we utilized established murine models of non-metastatic and metastatic breast tumors to identify bacteria capable of driving metastatic disease. We found several species of the *Bacillus* genus that were unique to metastatic tumors, and found that breast tumor cells cultured with a *Bacillus* bacterium isolated from metastatic tumors, *Bacillus thermoamylovorans*, produced nearly 3× the metastatic burden as control cells or cells cultured with bacteria from non-metastatic breast tumors. We then performed targeted metabolomics on tumor cells cultured with different bacterial species and found that *B. thermoamylovorans* differentially regulated tumor cell metabolite profiles compared to bacteria isolated from non-metastatic tumors. Using these bacteria, we performed *de novo* sequencing and tested for the presence of genes that were unique to the bacterium isolated from metastatic tumors in a patient population to provide a proof-of-concept for identifying how specific bacterial functions are associated with the metastatic process in cancer independent of bacterial species. Together, our data directly demonstrate the ability of specific bacteria to promote metastasis through interaction with cancer cells.

**IMPORTANCE:**

Metastasis is a major barrier to long-term survival for cancer patients, and therapeutic options for patients with aggressive, metastatic forms of breast cancer remain limited. It is therefore critical to understand the differences between non-metastatic and metastatic disease to identify potential methods for slowing or even stopping metastasis. In this work, we identify a bacterial species present with metastatic breast tumors capable of increasing the metastatic capabilities of tumor cells. We isolated and sequenced this bacteria, as well as a control species which failed to promote metastasis, and identified specific bacterial genes that were unique to the metastasis-promoting species. We tested for the presence of these bacterial genes in patient tumor samples and found they were more likely to be associated with mortality. We also identified enrichment of specific bacterial functions, providing insight into possible sources of bacteria-driven increases in the metastatic potential of multiple cancer types.

## INTRODUCTION

The ability of the microbiome to regulate tumorigenesis and disease progression has been demonstrated, not just in gut-localized tumors, but also distally in multiple cancer types (1–4). Several studies in pancreatic and liver cancer, for example, show that alteration of the homeostatic microbiome affects tumor growth (5, 6), and the use of human-to-mouse fecal transplant models in pancreatic cancer directly demonstrates the ability of the gut microbiome to promote or limit disease progression based on microbial composition (7). Additionally, several landmark studies in clinical melanoma show that patient outcome is influenced by gut microbial composition and can actually be improved through fecal transfer during anti-PD-1 immunotherapy (8–10). These studies reveal the existence of both anti-tumor and tumor-promoting functions controlled by microbiome composition, and demonstrate the clinical importance of understanding how the microbiome and specific bacterial populations control tumor growth in myriad cancer types.

Recent work in the field of breast cancer has revealed an association between different breast cancer types and composition of local bacterial populations. In the breast tissue, microbial composition varies between cancerous and normal adjacent tissue (11, 12), as well as between different tumor subtypes types (ER^+^, HER2^+^, and TNBC) (13–16). Studies in other cancer types such as colorectal cancer have elucidated the ability of specific bacterial populations to regulate tumor cell functions directly (3, 17, 18). Several recent studies also showed this dynamic can occur in breast cancer, as certain bacteria were shown to regulate either sheer stress resistance or β-Catenin signaling, and subsequently augment metastatic potential when directly introduced into tumor cells (19, 20). Despite this recent work, the functional role of bacteria within the breast tumor microenvironment (TME) and whether bacteria are relevant in tumor progression and patient outcome, remains to be determined. In addition, it remains to be determined if breast tumors of the same subtype with varying metastatic potential harbor unique bacterial populations, and if metastasis-specific bacteria preferentially increase metastatic potential compared to bacteria present in poorly metastatic tumors. Here, we sought to determine if, within a given subtype of breast cancer, tumors with varying metastatic potential contain different bacterial populations within the TME that contribute to differences in metastasis.

It is well-established that different types of breast cancer have differences in metastatic propensity, and that multiple components of the TME, such as hypoxia and nutrient composition, can regulate metastasis both positively and negatively. Because these environmental factors also regulate microbiome composition, we hypothesized that, within a given breast cancer type, tumors with varying metastatic potential and TMEs contain unique bacterial populations that contribute to differences in metastasis. To identify bacteria-specific effects on metastasis, we sought to identify, isolate, and utilize bacteria that are unique to metastatic disease to create a model system that demonstrates a level of causation beyond association with the TME. To do this, we used different models of either non-metastatic or highly metastatic TNBC, and tested for differences in bacterial regulation of disease progression. Through the use of multiple *in vivo* metastasis models, we demonstrated the ability of specific bacteria to promote metastatic disease. We then performed a retrospective clinical analysis and found the presence of corresponding bacteria and unique functional genes in human breast cancer. Taken together, our data demonstrate the ability of bacteria with different functional gene profiles to differentially regulate metastatic potential in breast cancer.

## RESULTS

### Metastatic and non-metastatic breast tumors harbor unique bacterial populations

We sought to compare two established triple-negative breast cancers with as few differences as possible outside of their ability to form metastatic disease. Based on these criteria, we established *in vivo* mouse tumor models using the highly metastatic 4T1, and the non-metastatic 67NR breast cancer cell lines that were originally derived from the same spontaneous BALB/c mouse mammary tumor (21, 22). These tumors have identical origins but vary significantly in their ability to form metastases (23, 24), and thus provide a suitable model for our proposed microbial study. Using these two cell lines, we sought to identify differences in intratumoral microbiome composition, and to isolate bacteria for direct analysis of bacteria-driven metastasis.

**Fig 1:**
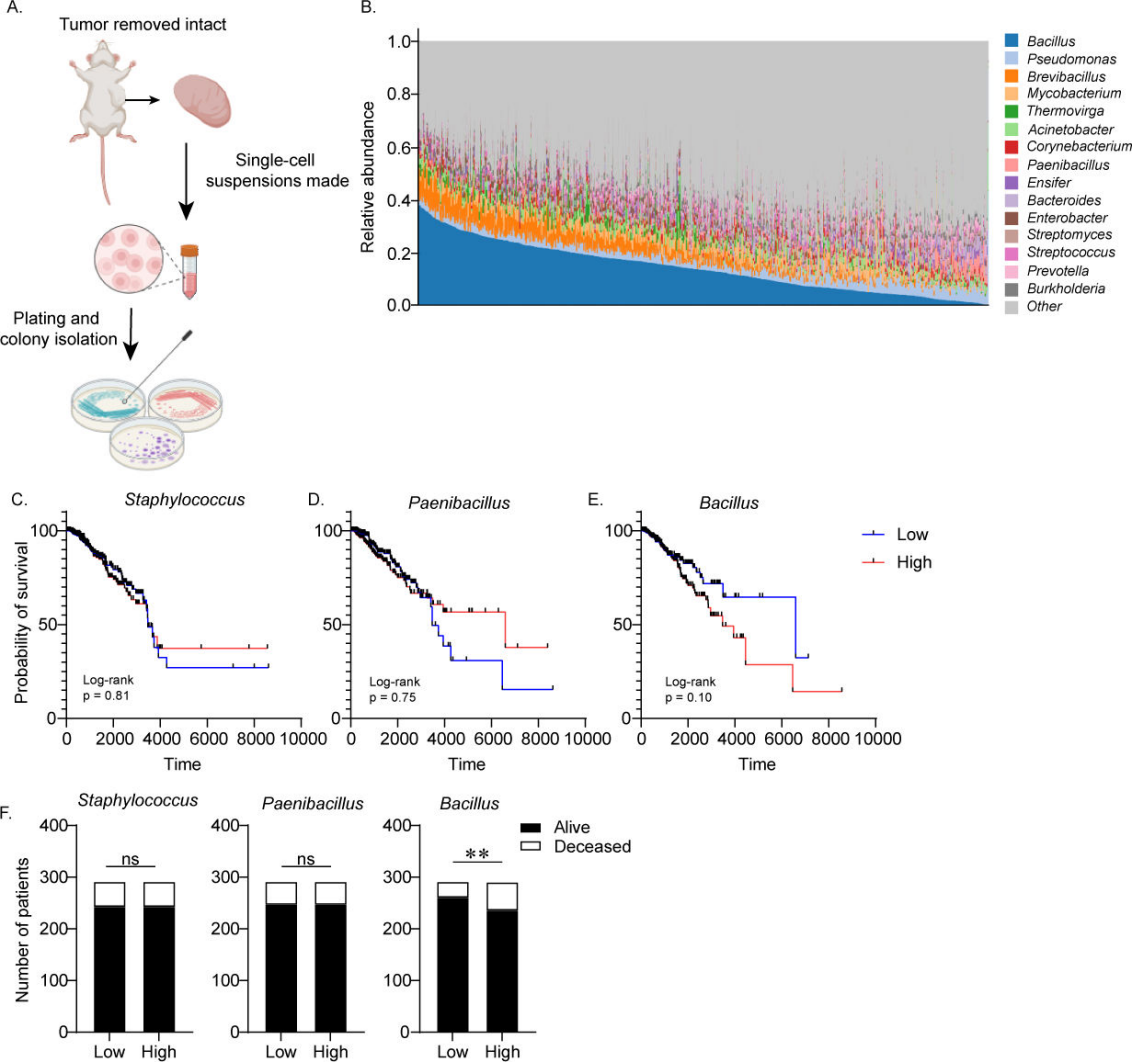
Metastatic and non-metastatic breast tumors harbor unique bacterial populations present in patient breast cancer samples. (**A**) Schematic depicting bacterial isolation from metastatic 4T1 and non-metastatic 67NR tumor tissue. (**B**) Genus-level data for intratumoral microbial composition of breast cancer (all types) patient samples available on the BIC database (21) shows the most abundant bacterial genera in human breast cancer. (**C–E**) *Staphylococcus, Paenibacillus*, and *Bacillus* abundances were determined for each patient and Kaplan-Meier analysis was performed on patients in the lowest and highest quartiles of *Bacillus* abundance. (**F**) Outcome data were compared between patients in the lowest and highest quartiles of abundance of the specified bacteria and Fisher’s exact test was used to compare the incidence of death between the two groups (**F**) with data for all patients summarized in Table 2. Data are presented as mean ± SEM. **P < 0.01.

To do this, 4T1 or 67NR tumors were inoculated orthotopically into BALB/c mice. When tumors reached an established volume, they were surgically removed intact and single-cell suspensions were made and plated onto bacterial growth media (Fig. 1A). We then isolated single bacterial colonies to produce bacterial stocks and performed Sanger sequencing and 16S alignment via BLAST to determine if metastatic tumors harbored unique bacterial populations compared to non-metastatic tumors (16S sequences used for BLAST alignment are available as fasta files in Data Set S1 (DOI: 10.5281/zenodo.11398890). While not a full characterization of intratumoral bacterial composition, this approach enabled us to identify viable bacteria and isolate those that could be used for additional investigation of their ability to interact with tumor cells. Interestingly, we saw high overlap at the genus level as both tumors demonstrated a high prevalence of bacteria of the *Bacillus* genus, as well as other members of the *Bacillota* (renamed from *Firmicutes* in 2021) phylum (Table 1), suggesting the TME of breast cancer provides an ideal growth environment for these bacteria. While Sanger sequencing a portion of the 16S gene does not provide definitive species classification, analysis of top hits suggests that, despite genus-level conservation, there was actually no overlap at the species level between *Bacillus* isolated from non-metastatic and metastatic disease (Table 1).

**TABLE 1:** Tumor-resident viable bacteria^*a*^

Source	Genus	Species hits (top 3)	Corresponding alignment (%)
4T1 1° tumor	*Bacillus*	***nealsonii****/alba/*sp. M-B	99.72/99.65/99.65
	*Bacillus*	***thermoamylovorans****/hisashii/*sp. B3	99.27/99.27/99.20
	*Paenibacillus*	sp. CCGE3113*/phoenicis/*sp. Q1	99.71/99.64/99.57
	*Paenibacillus*	*lautus*/sp. EZ-K15/sp. A4p128	99.86/99.79/99.65
	*Kocuria*	*indica/*sp. KD4*/marina*	100/100/100
	*Staphylococcus*	*epidermis*	99.60
4T1 Met	*Bacillus*	*nealsonii/**circulans**/alba*	99.52/99.51/99.51
	*Bacillus*	*thermoamylovorans*/sp. B3/sp. 1268	99.36/99.36/99.36
67NR 1° tumor	*Bacillus*	**PCWCS16**/*subterraneus*/sp S22906	99.79/99.79/99.71
	*Bacillus*	***subterraneus***/sp. S13_27F/sp. BCw063	100/99.86/99.86
	*Paenibacillus*	*lactis/*sp. 23B2.2/*ihbetae*	98.20/98.0/98.0

^*a*^ Sanger sequencing and BLAST alignment results for individual bacterial colonies isolated from 4T1 or 67NR primary (1°) tumors or 4T1 axillary lymph node metastases (Met). Individual colonies were collected and sequenced after plating on bacterial growth media and individual colony isolation was performed. Genus and species designation is for top hits based on alignment percentage. For *Staphylococcus*, all hits aligned to the same species. Bolded names are those used to differentiate *Bacillus* isolates. Genus and species designation is for top hits based on alignment percentage. When we performed similar bacterial culture analysis using 4T1 tumors isolated from antibiotic-treated mice we were unable to produce any bacterial colonies (*n* = 4).

Previous clinical studies have shown that *Bacillus* are enriched in cancerous tissue compared to adjacent normal tissue (12), and bacterial cultures derived from breast cancer patient samples also showed the presence of several *Bacillus* species (25). Given this recent clinical precedent, we analyzed previously curated TCGA miRNA sequencing data compiled on the Bacteria In Cancer (BIC) Database (26). We performed meta-analyses using the BIC database (26) focusing specifically on breast cancer patients (complete data used for analysis available in Data Set S2; DOI: 10.5281/zenodo.11398890). When analyzing the overall abundance of all bacteria in breast cancer patient samples, we found that the *Bacillus* genus was actually the most common, highly abundant genus in breast tumor tissue (Fig. 1B). We then performed the Kaplan-Meier analysis of overall survival based on abundance. In addition to *Bacillus* abundance, we also looked at overall survival compared to *Paenibacillus* and *Staphylococcus* abundance as defined by quartiles based on bacterial abundance level. Minimal association was found between overall survival and bacterial presence for both *Staphylococcus* and *Paenibacillus* (*P* value over 0.70 for both), and for both genera, the low abundance quartile reached a lower overall survival than the high abundance quartile. The *Bacillus* genus, however, actually showed the opposite trend, where the high abundance quartile was more strongly associated with reduced survival time than the low abundance quartile (Fig. 1C through E). While not reaching the level of statistical significance, this trend was notably stronger than what was observed for the other bacterial genera. Additionally, incidence analysis performed for all three genera revealed that, while no difference was seen based on *Staphylococcus* or *Paenibacillus* abundance, patients with a high *Bacillus* abundance had a significantly higher likelihood of succumbing to the disease, and demonstrated roughly half the median survival time compared to patients with low *Bacillus* abundance (Fig. 1F; Table 2). This was the case despite the fact that the low abundance group had ~40% more patients with stage III or IV disease (Table 2). To determine if abundance was associated with a particular stage of disease we assessed relative abundance across the stages and observed no significant differences (Fig. S1A) or associations between relative abundance and disease stage.

**TABLE 2:** Outcome data for BRCA patients^*a*^

Category	Metric	Low *Bacillus*	High *Bacillus*
Patient outcome	Median survival	6,593 days	3,472 days
	Censored	262	238
	Deaths	30	54
	Odds ratio	0.5047	1.982
Disease stage at collection (#patients)	Stage 1	45	55
	Stage 2	151	157
	Stage 3/4	80	57
	NA	13	20
	Total	289	289

^*a*^ Outcome data were compared between patients in the lowest and highest quartiles of abundance of *Bacillus* and Fisher’s exact test was used to compare incidence of death between the two groups. Outcome data were analyzed for all patients and broken down by disease stage as well.

### *Bacillus* species differentially affect tumor cells in culture

**Fig 2:**
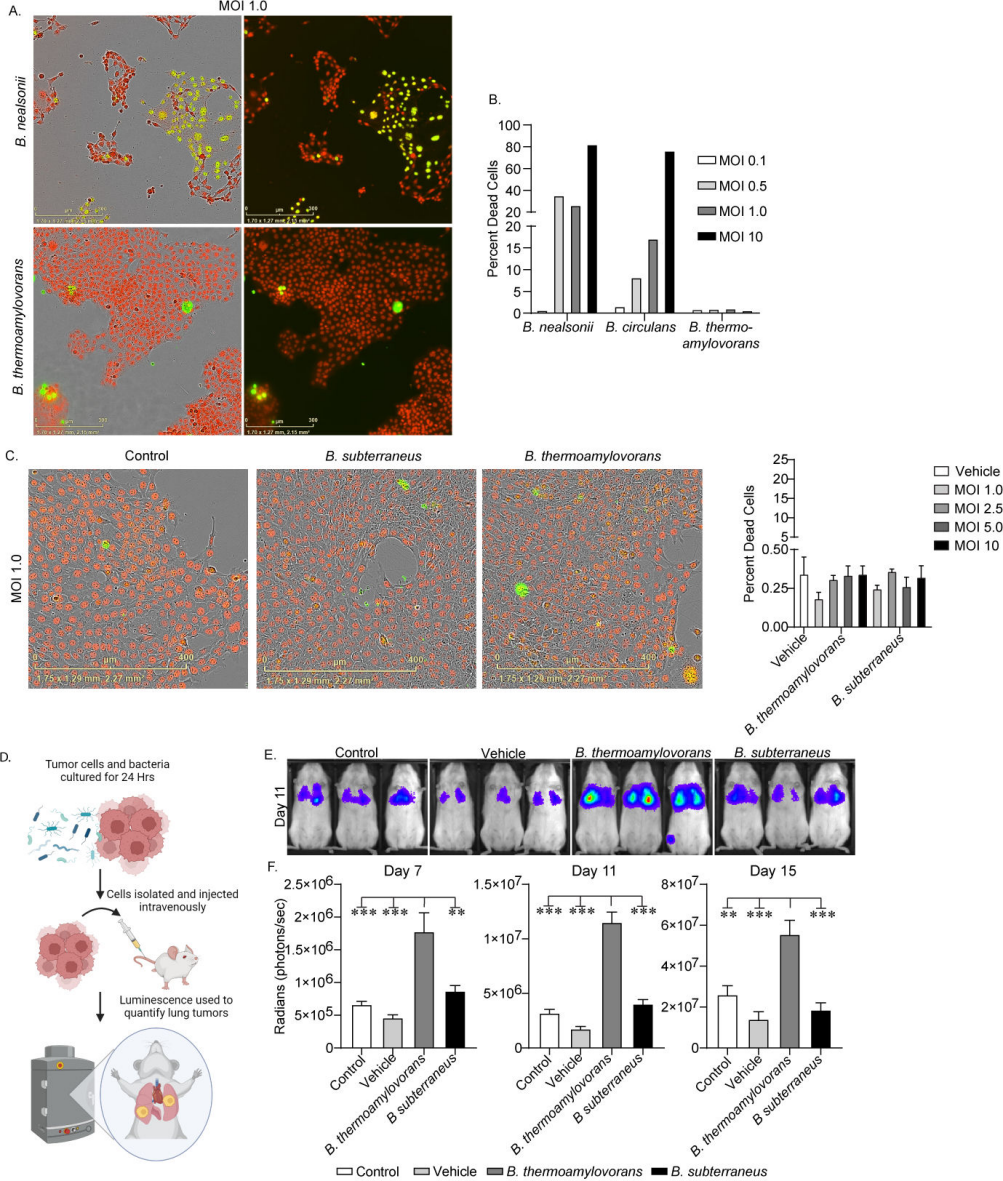
*Bacillus* species differentially affect tumor cell metastatic potential. (**A and B**) 4T1 tumor cells were co-cultured with different bacterial species at indicated MOIs for 24 h (Fig. S2E) (designations based on BLAST alignments). Cultures were then stained with HCS nuclear mask (red) to identify total cells and Sytox Green to identify dead cells (shown co-stained in yellow) and imaged via Incucyte. Representative images of viable cultures and cultures exhibiting widespread cell death are shown (**A**) and total and dead cells were quantified (**B**) to determine the percent dead cells (images and histograms representative of four independent experiments). (**C**) 4T1 tumor cells were co-cultured with indicated bacterial species isolated from either 4T1 or 67NR tumors across a range of bacteria concentrations for 24 h. Cultures were then stained and imaged and cells were quantified as described above (*n* = 3, representative of three independent experiments). (**D**) A schematic depicting experimental metastasis methodology is shown. (**E and F**) Luc+ 4T1 tumor cells were cultured without bacteria (control or FAB vehicle matched to bacterial MOI) or with indicated bacterial species at an MOI of 1.0 for 24 h. Bacteria were then washed away and cells were harvested and injected through the lateral tail vein into NSG mice. Lung metastases were visualized via bioluminescent imaging at indicated time points and metastatic burden was quantified (*n* = 6 per group). Data are presented as mean ± SEM. *P < 0.05, **P < 0.01, and ***P < 0.001.

Using our tumor-isolated *Bacillus* species, we next sought to establish a model system to directly test for potential metastasis-promoting effects of bacteria isolated from the TME. Our data identified several *Bacillus* species that were isolated from our model of both metastatic and non-metastatic breast cancer (Table 1). While Sanger sequencing results do not provide definitive species identities, we first sought to determine if these bacteria may be capable of regulating tumor cell function before proceeding with full genomic sequencing and characterization. We first attempted to culture these bacteria *in vitro* and observed that growth patterns were maintained when bacteria were cultured in varying growth media, including traditional bacteria growth media fastidious anaerobe broth (FAB), and standard tumor cell culture media DMEM + FBS (Fig. S2A through D), suggesting these bacteria could survive and proliferate normally in tumor cell culture conditions. We next performed co-culture experiments (Fig. S2E) initially with the *Bacillus* species and 4T1 tumor cells. While some of the species isolated from 4T1 tumors induced widespread cell death (Fig. 2A, top), one species, *B. thermoamylovorans*, a facultative anaerobe that is typically found in food but has also previously been isolated from the gut of healthy donors (genome assembly numbers: ASM2769222v1 and ASM2055444v1 on NCBI Assembly database)*,* failed to kill the tumor cells (Fig. 2A, bottom), even at multiplicity of infection (MOI) that resulted in almost complete cell death with other species (Fig. 2B). We next performed the same co-culture screen using bacteria isolated from the non-metastatic tumors and identified a *Bacillus* species *B. subterraneus*, that also did not induce significant cell death upon co-culture with tumor cells (Fig. 2C), as no bacterial concentrations resulted in cell death of greater than 0.5%.

### *B. thermoamylovorans* specifically promotes metastatic disease

We next tested whether the two bacterial species had any effect on metastatic potential. We did this using an experimental metastasis (Fig. 2D) model and intravenously administering 4T1 cells that had been co-cultured with either *B. thermoamylovorans* or *B. subterraneus* prior to injection. Strikingly, we found that while *B. subterraneus* had no effect on metastasis, the metastatic TNBC-derived *B. thermoamylovorans* significantly enhanced metastatic tumor burden with only 24 h of co-culture with the tumor cells (Fig. 2E and F). We then repeated the experiment using a higher bacterial MOI and again found that compared to control cells, *B. thermoamylovorans* significantly enhanced metastatic tumor burden (Fig. S3A and B). This phenomenon was indicated both by bioluminescence and by manual counting of metastatic nodules following tumor resection (Fig. S3A and B). To determine if any bacteria were injected along with the tumor cells, we performed CFU assays on the cells after they had been processed and re-suspended for intravenous injection and found no detectable bacteria remaining in the cell suspensions prior to injection (Fig. S3C and E). This suggests the augmented metastasis burden was a result of changes in tumor cell function introduced during the 24-h co-culture.

**Fig 3:**
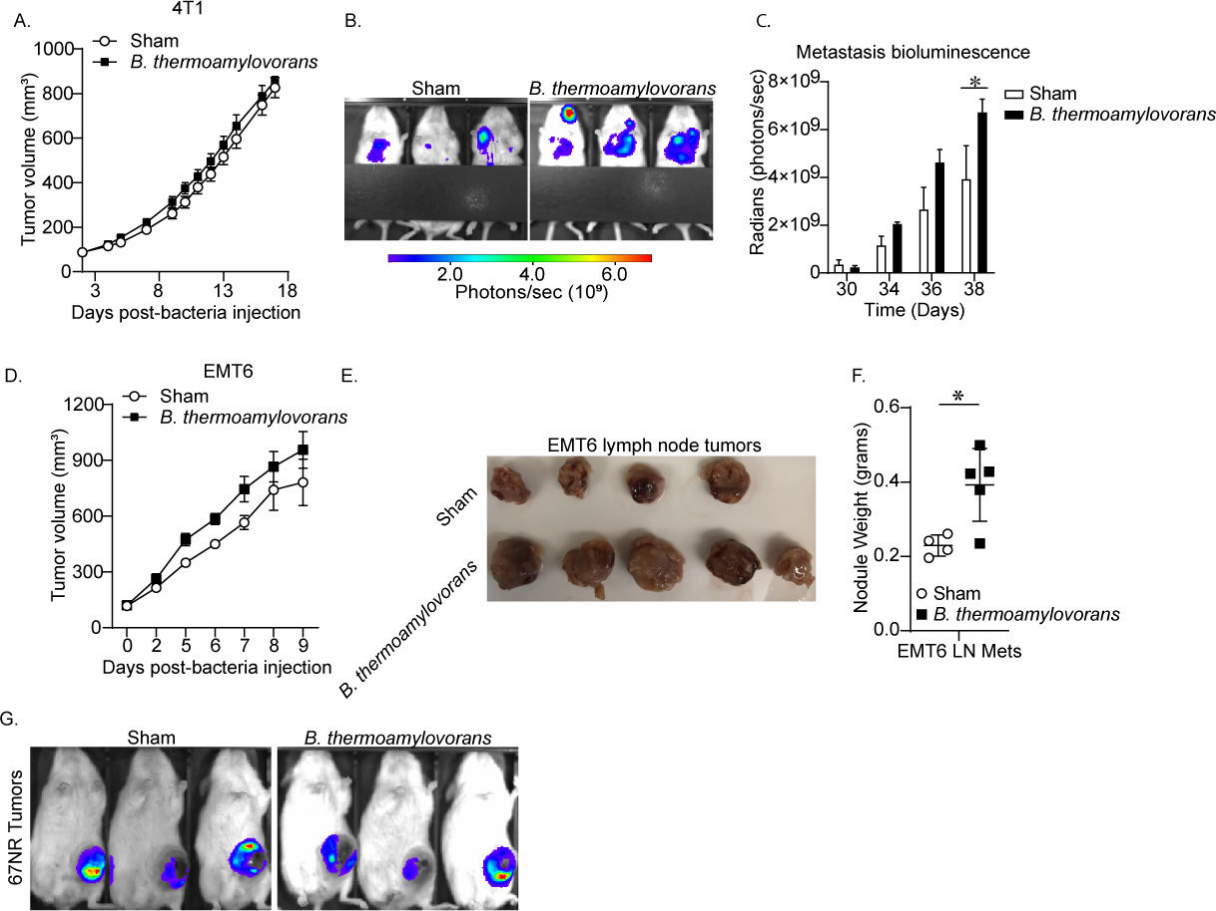
*B. thermoamylovorans* promotes metastatic disease following intratumoral injection. (**A–C**) NSG mice were inoculated orthotopically with luciferase+ 4T1 cells. When the average tumor volume reached 75 mm^3^, tumors were injected directly with saline (sham) or 5 × 10^4^ CFU *B. thermoamylovorans* 3× over the course of 1 week. (**A**) Primary tumor growth was quantified via caliper measurements and (**B and C**) lung metastases were imaged and quantified via bioluminescence (*n* = 3–4 per group). Tumor volume data are shown starting the day of the first injection. (**D–F**) EMT6 tumor-bearing mice were inoculated with *B. thermoamylovorans* using the same protocol as in panel **A**. (**D**) Primary tumor growth was quantified and (**E and F**) lymph node metastatic nodules were imaged and weighed (*n* = 4–5 per group). Tumor volume data are shown starting the day of the first injection. (**G**) NSG mice were inoculated orthotopically with luciferase+ 67NR tumor cells. When the average tumor volume reached 75 mm^3^, tumors were injected directly with saline (sham) or 5 × 10^4^ CFU *B. thermoamylovorans* 3× over the course of 1 week. Lung metastasis was evaluated via IVIS 2 weeks post-bacterial inoculation and no measurable signal above background was observed in the lung region of mice from either injection group. Data are presented as mean ± SEM. *P < 0.05.

To test the ability of *B. thermoamylovorans* to promote metastasis in established tumors, 4T1 or EMT6 tumor-bearing mice were inoculated intratumorally with either *B. thermoamylovorans* or sham/vehicle injections, and disease progression was monitored until mice reached clinical end point. We found that while *B. thermoamylovorans* had little effect on primary tumor growth, there was a significant increase in 4T1 lung metastases 2 weeks after bacterial injection (Fig. 3A through C). This experiment was repeated with the EMT6 tumor model, and we found axillary lymph node metastatic nodules isolated from EMT6 tumor-bearing mice were significantly larger from mice injected intratumorally with *B. thermoamylovorans* compared to vehicle control (Fig. 3D through F). We did not observe differences in lung metastases in this model, though we hypothesize this was due to the accelerated pace of disease progression. EMT6 tumor-bearing mice had to be euthanized only 2 days after the final bacterial injection due to reaching the clinical end point and were likely well into the end stage of disease progression during *B. thermoamylovorans* inoculation (Fig. S3E), making detection of any differences in lung metastases difficult with this model. We also performed similar experiments with 67NR tumor-bearing mice and found *B. thermoamylovorans* was not sufficient to cause metastasis in non-metastatic tumors (Fig. 3G).

### Metastatic and non-metastatic tumor-derived bacteria differentially regulate tumor cell metabolite profiles

**Fig 4:**
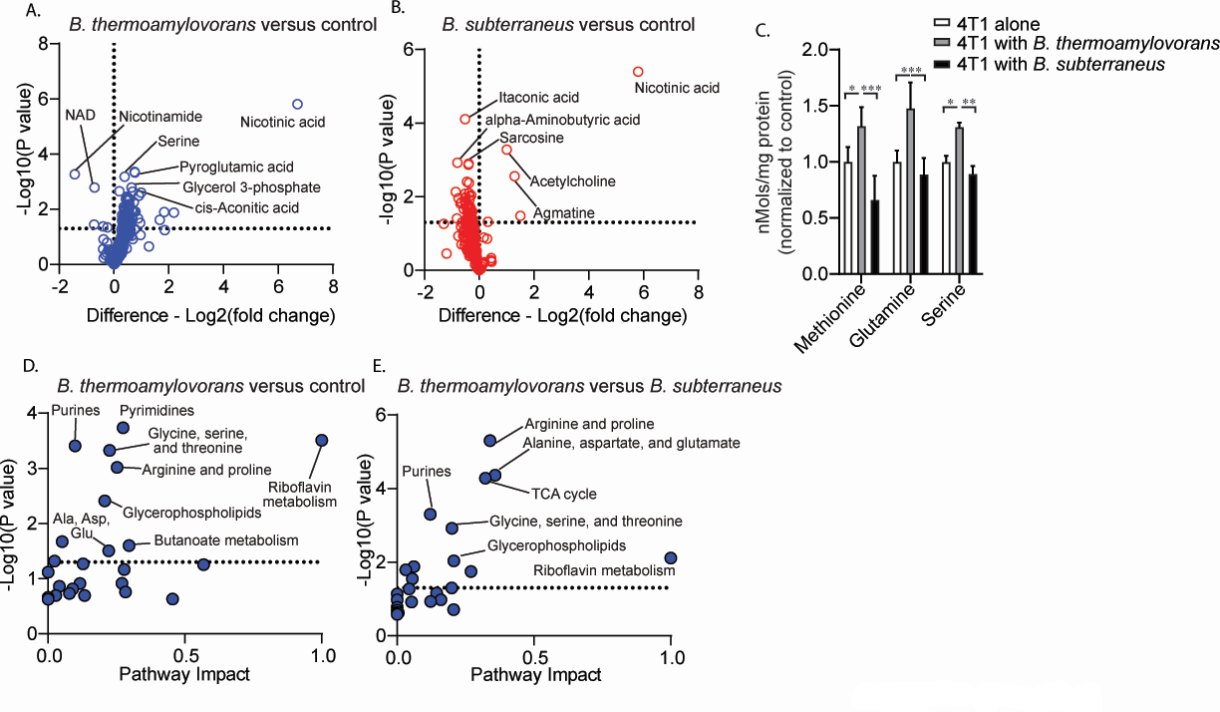
4T1 and 67NR-derived bacteria differentially regulate tumor cell metabolite profiles. (**A and B**) 4T1 cells were cultured for 24 h in normal culture media with either *B. thermoamylovorans* or *B. subterraneus*. Bacteria and culture media were washed after 24 h and metabolite extracts were collected from tumor cells and targeted metabolomics analysis was performed on glycolytic, redox, TCA cycle, nucleotide, and amino acid metabolites. Differences relative to control cells are shown for individual metabolites and are plotted against the results of multiple *t* tests performed on the individual metabolites. (**C**) Quantification of select amino acids from targeted metabolomics, full amino acid results available in supplemental tables. (**D and E**) KEGG pathway enrichment analysis was performed using MetaboAnalyst on metabolites found to be significantly different between the indicated groups. Raw *P* values are shown plotted against pathway impact calculated from pathway topology analysis (full analysis details included in analysis reports included with Data Set S3; DOI: 10.5281/zenodo.11398890). *N* = 4 for the control group and *N* = 3 for the bacteria-exposed groups. Dashed line on plots is indicative of a *P* value of 0.05. *P < 0.05, **P < 0.01, and ***P < 0.001.

We next tested for differential regulation of tumor cell function between the metastasis-promoting and control bacteria. Because metabolite-based signaling is one of the primary communication mechanisms between cells and bacteria, we performed targeted metabolomics analysis on metastatic TNBC cells that had been co-cultured with either bacterial species. We did this by culturing 4T1 tumor cells either alone as controls or with either bacterial species. We then removed culture media and extracellular bacteria to enable extraction specifically of intracellular metabolites. Targeted metabolite quantification revealed the most significant change compared to control cells was in levels of nicotinic acid and that this was the case following co-culture with either bacterial species (Fig. 4A and B). Full metabolite analysis, however, showed that co-culture of 4T1 cells with *B. thermoamylovorans* caused significant increases in the number of metabolites compared to control cells that were not seen following co-culture with *B. subterraneus* (Fig. 4A and B; Tables S1 to S3). These included several TCA cycle and nucleic acid intermediates, as well as a number of amino acids (Fig. 4A and B; Tables S1 to S3). Several amino acids have been shown to be critical for metastasis both for biosynthetic purposes and as signaling molecules, and serine, methionine, and glutamine are among those that have been shown to drive metastasis in TNBC specifically (27–29). While not all amino acids were upregulated (Tables S1 to S3), we observed augmented levels of these established metastatic drivers following co-culture specifically with *B. thermoamylovorans* (Fig. 4C).

Beyond the individual metabolite level, we next performed pathway analysis using the metabolites that were enriched in the 4T1/*B. thermoamylovorans* co-cultures both compared to control cells and compared to cells cultured with *B. subterraneus* (Fig. 4D and E). Analysis was performed using the MetaboAnalyst (30) analysis tool to identify KEGG metabolic pathways that were over-represented in the enriched metabolite data sets. Full analysis methodology and enrichment results are described in the MetaboAnalyst analysis reports found in Data Set S3 (DOI: 10.5281/zenodo.11398890). We observed the enrichment of multiple metabolic pathways in 4T1 cells following co-culture with *B. thermoamylovorans* when compared to control cells or cells cultured with *B. subterraneus* (Fig. 4D and E). As expected based on individual metabolite levels, several amino acid metabolic pathways were activated in 4T1 cells following co-culture with *B. thermoamylovorans*. We also observed upregulation of purine and pyrimidine metabolism, which aligns with the increased presence of nucleic acid intermediates seen at the individual metabolite level. Overall these data demonstrate that bacteria with different functional gene profiles are capable of inducing unique metabolic changes in tumor cells.

### Metastasis-promoting *B. thermoamylovorans* harbors unique functional genes

**Fig 5:**
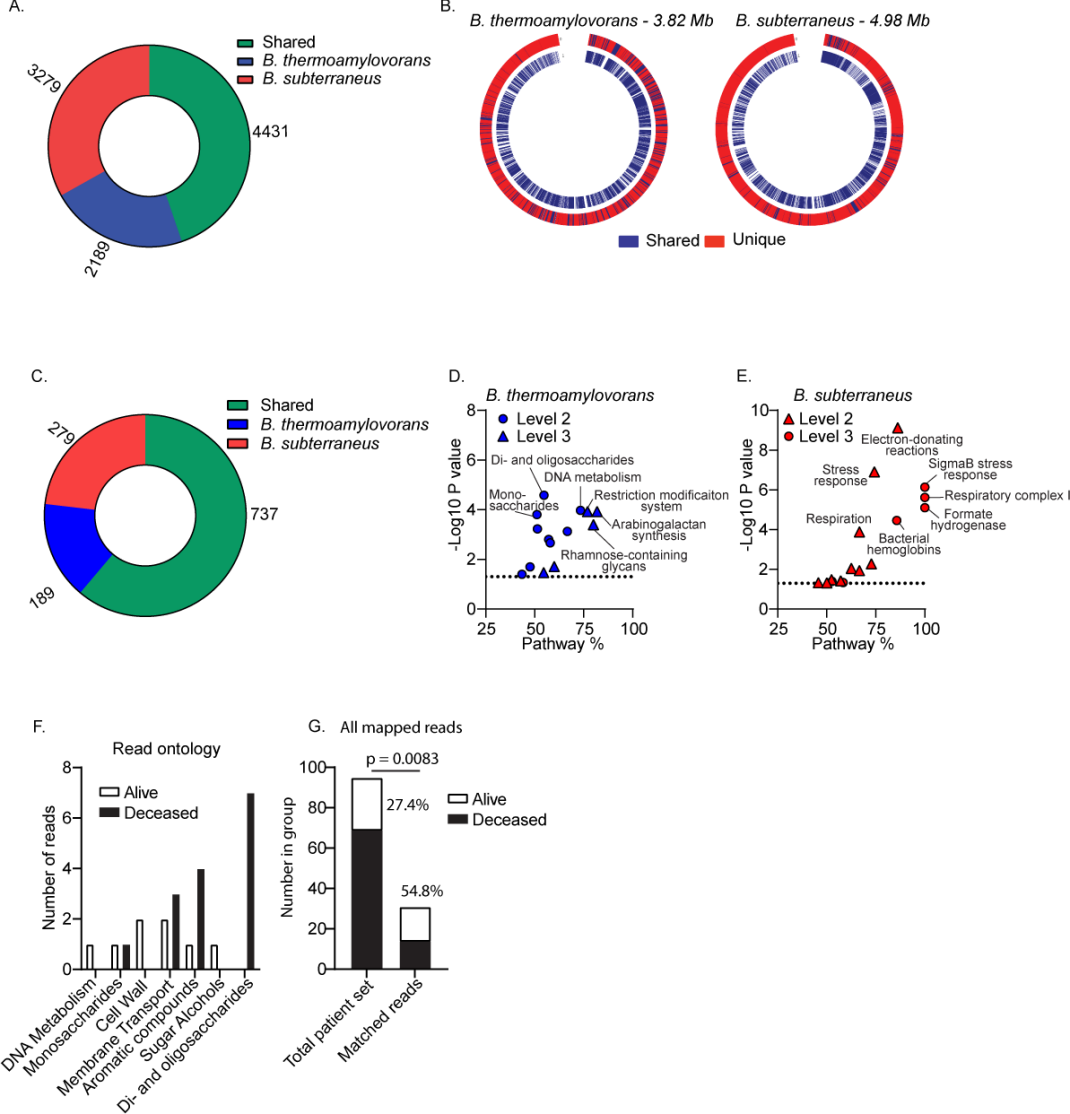
Presence and association with mortality of *B. thermoamylovorans*-specific genes. *De novo* sequencing and genome assembly were performed on *B. thermoamylovorans* and *B. subterraneus* as described in Materials and Methods. (A) Total unique or shared bacterial coding sequences (CDS) found between *B. thermoamylovorans* or *B. subterraneus* were quantified and a pan-genome was created (B) (Data Set S6, DOI: 10.5281/zenodo.11398890) to identify overlapping and unique features with the outer ring (red) showing the shared or unique features of the indicated bacterium and the inner ring (blue) showing the shared features found in the genome of the other species. (C) CDS were then mapped to functional genes using the seed database and total unique or shared functional genes found between *B. thermoamylovorans* or *B. subterraneus* were quantified after SEED mapping and annotation. (D and E) After SEED gene and category-level classification, Fisher’s exact test was used to identify pathways that were significantly enriched in *B. thermoamylovorans* or *B. subterraneus* based on the number of unique CDS present in a given pathway. Volcano plots show *P* value and percent of coding sequences in a given pathway that was unique to the specified bacterium. Dashed line is indicative of a *P* value of 0.05. Significant results are shown in Fig. S4 and complete results are available in Data Set S7 (DOI: 10.5281/zenodo.11398890). (F–H) Bacterial DNA sequences from SEED pathways that were demonstrated to be significantly enriched in *B. thermoamylovorans* or *B. subterraneus* (Fig. S4) were used to map bacterial sequencing data obtained from whole-genome sequencing analysis performed on TNBC patient biopsies. Unmapped reads from patient samples were aligned to the unique genome feature sequences from *B. thermoamylovorans* or *B. subterraneus*, and reads that mapped to a given bacterial DNA sequence and the associated functional gene were identified. (F) Ontology of reads from alive and deceased patients (Level 2 classification) was compared to determine the functional overlap of mapped reads. (G) Histograms comparing the source of mapped reads to the makeup of the overall patient data set reveal preferential association with deceased patients for genes from the metastasis-promoting bacteria. Fisher’s exact test was used for statistical comparisons and *P* values are shown directly.

We next sought to identify functional genes that were unique to *B. thermoamylovorans* compared to *B. subterraneus* in order to better characterize the bacterium capable of promoting metastatic disease. To do this, we performed *de novo* sequencing on both *B. thermoamylovorans* and *B. subterraneus* and annotated their entire genomes (Fig. 5A through C). Full assembly, classification, genome analysis, and annotation results are available in Data Sets S4–S**7** (DOI: 10.5281/zenodo.11398890). Classification analysis revealed both bacteria could be classified to at least the genus level with minimal contamination (<3% based on CheckM analysis tool [31–33]), and the 4T1-derived bacterium was indeed classified as *B. thermoamylovorans*. At the genus level, the 67NR-derived bacterium classified as a member of the *Bacillus* genus that was recently reorganized into a *Bacillus* subset called *Mesobacillus* (34), and at the species level, the closest placement taxonomy was indeed *Mesobacillus subterraneus*, as expected.

The majority of coding sequences (CDS) and functional gene classifications identified were shared between the two bacterial species (Fig. 5A through C), likely reflecting their shared identity at the genus level. Each species, however, also harbored thousands of unique CDS that corresponded to hundreds of unique functional genes (Fig. 5A through C). We performed enrichment analysis on genome sequences that were unique to either the metastasis-promoting or the control bacteria. To do this, we looked at genome sequences that were unique to a given species, and performed statistical tests to identify functional pathways that were over-represented within these unique genomic sequences. After correcting for multiple comparisons, we identified multiple functional pathways that were enriched in *B. thermoamylovorans* compared to *B. subterraneus*. These included several core metabolic pathways (di- and oligosaccharide metabolism, DNA metabolism), as well as multiple pathways associated with metabolite transport and utilization (Fig. 5D and E; Fig. S4). Several functional pathways were over-represented within the unique genomic sequences found in *B. subterraneus* as well, with the primary difference being a prevalence of CDS associated with the capacity for bacterial respiration and an increased presence of CDS classified as bacterial stress response genes that were not found in *B. thermoamylovorans* (Fig. 5D and E; Fig. S4).

Interestingly, multiple metabolites and pathways associated with di- and oligosaccharide metabolism and DNA metabolism have demonstrated roles in cancer. This includes lactate (35), which is the primary metabolite produced in bacterial glycolysis downstream of oligosaccharide metabolism and has been shown to promote both cancer development and metastasis (36, 37). Regarding DNA metabolism, nucleotide metabolism and synthesis pathways have been shown to be critical for tumor cell metabolism and tumor growth (38), and in breast cancer specifically, activation of *de novo* nucleotide synthesis has actually been shown to drive cancer cell stemness and promote metastasis (39). This suggests a potential association between these genes and the enhanced metastasis seen following co-culture with *B. thermoamylovorans*, though further study is needed to define these relationships.

### Presence and association with mortality of *B. thermoamylovorans*-specific genes

Different bacterial within the same microenvironment may exhibit functional redundancy where different species can have similar effects on their surroundings. Because of this, we sought to characterize the intratumoral microbiome in terms of bacterial functional genes rather than specific genera or species. As a proof of principle for the analysis of clinical associations with bacterial functional pathways, we next tested for the presence of pathways that were enriched in our metastasis-promoting bacterial model in patient breast cancer samples. To do this, we used whole-genome sequencing results from TNBC patient samples compiled as part of the B-PRECISE biorepository (S. Aparicio, BCCRC) (40). We obtained whole-genome sequencing data from tumor tissue and matched normal tissue biopsies from 95 TNBC patients (40). To identify bacteria-derived sequences, we then removed all reads that were part of the human genome to eliminate any sequencing data derived from patient DNA. We then took the remaining unmapped reads from each sample and mapped them against the unique genomic features identified in our models of metastasis-promoting and control bacteria using the actual bacteria-derived DNA sequences. Because the primary goal was to test for the presence of genes found in *B. thermoamylovorans* or *B. subterraneus*, we used the CDS from any gene set that showed enrichment before multiple comparisons correction, leading to a set of 121 CDS from *B. thermoamylovorans* and 133 CDS from *B. subterraneus* (Fig. S4).

Despite the narrow data set resulting from using mammalian sequencing data and the use of the actual CDS from our two bacterial models as reference data sets, we were still able to identify multiple reads across multiple patient samples that actually mapped to our bacterial gene sets. A total of 31 individual reads derived from the patient samples mapped to various CDS that were specific to *B. thermoamylovorans,* while only 2 reads mapped to sequences that were specific to *B. subterraneus* (full results available in Data Set S8, DOI 10.5281/zenodo.11398890). As we were interested in the functional profile of these mapped reads, we then compared the function of the CDS derived from alive or deceased patients. We found overlap in several SEED functional classifications (Fig. 5F); strikingly, though, we found that the reads from the deceased patients most often mapped to the di- and oligosaccharide pathway (the most enriched pathway in *B. thermoamylovorans*, Fig. S4), and none of the reads derived from the alive patients mapped to this pathway.

As a proof of principle, we also tested for any association with mortality to determine the feasibility of using bacterial functional genes as indicators of disease outcome by looking at the patient and tissue source of individual reads. We then used Fisher’s exact tests and calculated odds ratios to compare the odds of a given mapped read being from a deceased patient to the likelihood of a given patient in the entire data set being from the deceased group (Fig. 5G; Table 3). We evaluated the read set and found that the majority of the reads were actually from deceased patients despite these patients making up only 27% of the total population (Fig. 5G) (full patient outcome data can be made available upon request). We analyzed tissue sources by evaluating reads from both tumor and normal tissue samples. Interestingly, we found that reads from both normal and tumor tissue sources were significantly more likely to come from patients in the deceased group compared to the alive group based on Fisher’s exact tests and odds ratios calculations (Table 3). We then specifically looked at reads from normal tissue where no other reads were present in tumor samples from the same patient to see if any association changed when reads were present only in normal tissue (Table 3). This analysis identified the only instance where the odds ratio matched what would be expected based on patient population statistics, as 25% of these “normal only” reads came from the deceased group (27% of the total patient population) (Table 3). While not sufficiently powered for a formal correlation studies, these analyses reveal a trend in which certain bacterial functions, in particular certain bacterial metabolic pathways, can associated with patient outcomes, and provide a proof-of-concept for evaluating the role of intratumoral bacterial functions without requiring species or even genus-level classification.

**TABLE 3:** Comparison of reads mapped to *B. thermoamylovorans^a^*

Metric	Outcome data		Normal tissue reads		Tumor tissue reads		Normal only reads	
	Alive	Deceased	Alive	Deceased	Alive	Deceased	Alive	Deceased
Number	69	26	7	7	7	10	6	2
Percent (%)	72.6	27.4	50	50	41.2	58.8	75	25
Odds ratio	–		2.65 (*P* = 0.1182)		3.79 (*P* = 0.0212)		0.88	
	–		3.22 (*P* = 0.0083)				–	

^*a*^ Mapping results for data presented in Fig. 5 are summarized in table format, which shows patient statistics and read statistics based on tissue source and patient outcome. Normal and tumor tissue were considered separately for odds ratio calculations, and the combined calculation is for normal and tumor tissue. The final column shows reads derived from normal samples where no reads were obtained from the corresponding tumor samples. Odds ratio calculations were comparisons of the likelihood a mapped read was from a decease patient compared to likelihood of a given patient in the entire data set being deceased. – indicates odds ratio calculations not performed, as data in the “Outcome data” column are descriptive and no comparisons are made and as data in the “Normal only reads” column are fully analyzed with the single calculation shown.

## DISCUSSION

We sought to determine if bacteria within the TME of metastatic or non-metastatic tumors may differentially contribute to metastatic disease. While *B. thermoamylovorans* and *B. subterraneus* are not the only bacteria present in the TME, as a proof of concept, we used these two species as representative models for testing the metastasis-promoting functions of bacteria that reside in the TME of metastatic or non-metastatic disease. In doing so, we identified a bacterial species that was uniquely capable of augmenting metastatic disease and found a preferential association with mortality for the *B. thermoamylovorans*-specific gene set in patient samples. We also observed unique changes in tumor cell metabolite profiles following co-culture with the metastasis-promoting bacteria. While clinical studies have previously identified *Bacillus* bacteria as being enriched in breast cancer, our data provide novel insight into a potential role for these bacteria, and the functional genes present in metastasis-promoting bacteria in general, in regulating disease progression.

While overall disease progression results from previous studies in mice assessing the role of bacteria in breast cancer are similar to our findings here (19), the previous study showed little overlap with our results when culturing and identifying bacteria from the murine TNBC TME. Although a difference in breast cancer type is one possible explanation, the difference in species identified is also potentially due to experimental design in that the culture experiments in the previous study were designed to isolate and culture intracellular bacteria, specifically (19). The ability of extracellular bacteria to alter host cell function has been well-established in many settings ranging from homeostasis to pathogenic infection to immune regulation (41–44). In breast cancer specifically, the extracellular bacteria *B. fragilis* was shown to alter tumor cell function through secretion of *B. fragilis* toxin rather than through an intracellular mechanism (20), and our data here reveal a role for extracellular bacteria in promoting metastasis as well.

Our genomic analysis identified several functional pathways that were unique to our metastasis-promoting bacterial model. While patient numbers limited our statistical analysis, the fact that nearly 50% of the genes from deceased patients were members of the di- and oligosaccharide pathway, and that none of the genes from living patients were, suggests an association between this bacterial functional pathway and mortality. Members of this pathway identified in patient samples primarily function to fuel glycolysis through different substrates including galactose and maltose (45, 46), suggesting bacteria within tumors from the deceased population may exhibit enhanced glycolytic activity. This is notable considering the differences in pathway enrichment in *B. thermoamylovorans* and *B. subterraneus,* where *B. subterraneus* exhibited significant enrichment of respiratory pathways as opposed to bacterial glycolysis. This suggests a model wherein non-metastatic and metastatic breast tumors, even of the same type, may differentially shape bacterial composition through regulation of the nutrient environment. Given that lactate is the primary product of bacterial glycolysis (35), in particular, bacterial fermentation in low oxygen conditions as may be found in the TME, and the critical role lactate has in tumor development and metastasis (36, 37), these data also suggest a model wherein byproducts of bacterial glycolysis may in turn drive disease progression. Though further research is needed to validate the importance of any functional genes described in this study, the functional specificity of metabolic pathways seen in deceased patients warrants further investigation of bacterial metabolism-based regulation of tumor cell functions.

Regarding *B. thermoamylovorans*-specific changes in tumor cell function, several of the metabolic pathways enriched by *B. thermoamylovorans* have previously been associated with metastatic disease. For example, a recent study of TNBC patient samples (28) showed that compared to primary tumor tissue, tumor cells collected from three different metastatic sites (liver, lung, and brain) showed enrichment of several amino acid metabolism pathways that were also enriched in 4T1 tumor following co-culture with *B. thermoamylovorans* and not *B. subterraneus*. In addition to amino acid metabolism pathways, our data were also congruent with previous work showing activation of nucleotide synthesis pathways, specifically pyrimidine and purine metabolism (38, 39, 47). Nucleotide metabolism has been shown to contribute to the disease progression of multiple cancer types, and, in TNBC specifically, multiple pre-clinical studies have shown that *de novo* nucleotide synthesis can activate several tumorigenic functions (39, 47). Similar to amino acids metabolism, activation of these nucleotide synthesis pathways was associated with augmented metastatic potential in that these changes were exclusive to the metastasis-promoting *B. thermoamylovorans* bacteria. Though further study is necessary to determine the role of individual metabolites in metastasis specifically in the context of *B. thermoamylovorans* exposure, the metabolic activation seen following co-culture with *B. thermoamylovorans* suggests a pro-metastatic phenotype. Beyond the metabolite data, the fact that no changes in metastasis were seen following co-culture with *B. subterraneus* suggests that the specific effects on tumor cell function induced by our metastasis-promoting bacterium model, rather than those induced simply by the presence of bacteria, are indeed driving metastatic disease.

While these functional gene profiles were established in *B. thermoamylovorans* by comparisons to a *B. subterraneus* control bacterium, we hypothesize bacteria with similar functional profiles would also be capable of promoting metastatic disease. Our data here provide definitive evidence that bacteria that are similar based on genus-level classification can have significantly different effects on metastatic potential. Because bacteria of different species can exhibit functional redundancy, we believe it is critical to consider bacterial functional genes associated with disease progression and mortality. Identifying bacterial functions associated with disease progression also avoids potential composition differences that may simply be a result of geographic location or race and gender differences. This pro-metastatic bacterial gene set therefore represents a novel, and direct analysis of how bacteria may promote metastatic disease in clinical TNBC, and a tool that could be used to identify metastasis-promoting bacterial genes in the patient population independent of specific bacterial species.

### Limitations of the study

While our data clearly demonstrate the ability of specific bacteria to promote disease progression in metastatic breast cancer, further study is required to determine the mechanism driving this augmented metastasis phenotype. Our study is also limited to analysis of only those bacteria we were able to culture directly from orthotopic tumors, and other bacteria that were not culturable using our lab’s methods may also be playing a role in regulating disease. We believe our study provides a model for using analysis of bacterial functional genes rather than species identity in determining how the microbiome contributes to tumor progressio. However, the identity of the critical functional genes remains an open question even with our clinical analyses here. It would also be interesting to expand our clinical analysis of bacterial functional genes to include overall survival and in particular distal disease recurrence. However, those questions are beyond the scope of this study as metagenomic sequencing data on new tissue samples with significantly improved depth of bacterial reads would likely be required.

## MATERIALS AND METHODS

### Mice, cell lines, and reagents

Wild-type BALB/c mice were purchased from Jackson Laboratory (Bar Harbor, ME, USA). Mice were housed for 1 week in the BC Cancer Research Centre Animal Resource Centre (ARC) prior to the initiation of vehicle or antibiotic treatment. NOD/SCID IL2Rγ^−/−^ (NSG) mice were obtained through an “in-house” breeding program using breeders from Jackson Laboratories. The murine mammary adenocarcinoma 4T1 (CRL-2539), 67NR (CVL_9723), and EMT6 (CRL-2755) were purchased from the American Type Culture Collection and validated by STR analysis. All cell lines were maintained in DMEM (Gibco, #11995-065) plus 10% FBS (Gibco, #12483020) and 1× nonessential amino acids (Gibco, #11140-050). Cell lines were transduced via lentivirus to stably express firefly luciferase and expression was maintained through puromycin selection and luciferase expression was verified both before and after orthotopic inoculation. XenoLight d-Luciferin (#122799) was purchased from PerkinElmer (Waltham, MA, USA).

### Cell culture

For 4T1, 67NR, and EMT6 cell cultures, the cells were incubated in a humidified incubator at 37°C with 5% CO_2_. Cell lines were routinely tested for mycoplasma contamination using the MycoAlert Mycoplasma Detection Kit (Lonza, Mississauga, ON, Canada). Cell lines used in the manuscript have been authenticated using short tandem repeat DNA profiling (DNA fingerprinting) by a commercial testing facility (Genetica, Burlington, NC, USA). Cell lines were maintained through serial passage at 90% confluency with fresh media and puromycin in the case of luciferase-positive cells. The cells were used for *in vivo* tumor growth assays at 70–80% confluency. For bacterial culture, isolated bacteria were stored as glycerol stocks and freshly streaked onto fastidious anaerobe agar plates for each experiment. Individual colonies were cultured overnight in liquid cultures and sub-cultures were prepared on the day of each experiment to ensure bacteria were actively growing at the time of exposure to tumor cells.

### Bacterial culture and colony isolation

Primary tumors or metastatic nodules were removed from BALB/c mice using the sterile tip technique. Samples were placed in reduced phosphate-buffered saline (PBS) and transported under low-oxygen conditions. Tissues were then washed 3× in a PBS bath to remove any extra-tumoral bacteria with the third wash being plated as a control alongside tumor suspensions. Suspensions were made by rough sectioning of tissue followed by mechanical digestion in PBS using granite bead tubes. Mechanically digested suspensions were then plated directly onto fastidious anaerobe agar or brain-heart infusion plates, or aliquoted into FAB or brain-heart infusion broth, and suspensions were then cultured either under normoxic or anoxic conditions. Plates with viable colonies were re-streaked and liquid cultures were plated onto agar plates and re-streaked until individual colonies could be isolated. Individual colonies were then made into glycerol stocks and stocks were sub-cultured for sanger sequencing of the 16S gene for taxonomic classification based on alignment score.

### Sanger sequencing

Individual bacterial colonies were selected and grown overnight in appropriate bacterial growth media. Bacterial cultures were then sub-cultured the next day by diluting 1:10 in bacterial growth media and growing bacteria were collected, washed 2× with PBS and pelleted. Bacterial pellets were then sent to Azenta Inc. (formerly Genewiz, Burlington, MA, USA) for Sanger sequencing using their standard 16S sequencing protocol in which a portion of the 16S gene is sequenced. If read length produced sufficient overlap, sequencing reads from forward and reverse primers were then aligned and edges trimmed. The resulting alignments were analyzed via BLAST against the NCBI Nucleotide collection database. Where reads were not of sufficient length for alignment, forward and reverse reads were analyzed and top overall hits from both analyses were included. Hits were sorted by alignment percentage to identify potential hits with the highest sequence similarity. Top three hits are shown, with the species name used to differentiate bacterial isolates used over the course of this study shown in bold.

### BIC database analysis

For BIC database, all breast cancer patients present in the database were selected and relative abundance data for bacterial composition was plotted for each patient. Patient data were not broken down by tumor type so all types of breast cancer were considered together. For all three bacterial genera analyzed, patients were sorted based on relative abundance. This was done separately for each bacterial species. Patients were then sorted into quartiles and patients falling in the highest and lowest quartiles were compared. Outcome data were used for Kaplan-Meier analysis and tabular data, while incidence analysis was used to determine odds ratio for survival outcomes.

### Bacteria/tumor cell co-culture

For co-culture experiments, 2 days prior to co-culture, tumor cells were seeded in 24-well plates and allowed to grow for 48 h. Fresh bacteria agar plates were also made using fastidious anaerobe agar (FAA) and allowed to grow overnight. Twenty-four hours prior to co-culture, individual bacteria colonies were selected for overnight culture in liquid FAB and tumor cell growth was maintained. On the day of co-culture, bacteria were sub-cultured in FAB and then diluted to indicated MOIs in standard tumor cell culture media. MOI calculations were based on cell counts in test wells and FAB alone was used as a vehicle control. Co-cultures were placed in a CO_2_ incubator for indicated time points prior to downstream analysis. This methodology was used for all bacteria/tumor cell co-culture experiments unless otherwise indicated.

### Animal experiments

For tumor inoculation, tumor cell lines were inoculated into 7- to 9-week-old female BALB/c mice. For orthotopic tumor growth experiments, 1 × 10^6^ cells/animal were implanted into the fourth left mammary fat pad and tumor growth was monitored via caliper measurements.

### *In vivo* experimental metastasis assays

For experimental metastase assays, tumor cells and indicated bacteria were co-cultured together as described for 24 h and bacteria were then removed as indicated in Fig. S3C and D. The cells were then harvested and 5 × 10^5^ tumor cells were inoculated intravenously through the lateral tail vein of NSG mice. IVIS measurements were taken at indicated time points beginning 4 days post-injection. Mice were euthanized at end point and whole lungs were resected for counting of visible nodules.

### Orthotopic metastasis assays

For intratumoral injection experiments, indicated cell lines were implanted orthotopically into NSG mice. Tumors were allowed to progress until average size reached approximately 75 mm^3^ and mice were sorted into matched groups based on tumor size. Mice were then injected intratumorally with 5 × 10^4^ CFU of sub-cultured bacteria in saline with matched FAB used as a vehicle control. Mice were injected 3× over the course of 7 days and tumor growth was monitored via caliper measurements over indicated time frames. IVIS measurements were performed as tumor volumes reached the experimental end point. For animals injected with EMT6 cells, axillary lymph node tumor nodules were resected intact at the end of the experiment timeline and weights were collected for these metastatic nodules.

### *In vivo* bioluminescent imaging

Luciferin was dissolved in saline solution at 15 mg/mL and 10 µL/g body weight was injected intraperitoneally. Ten minutes post-injection, bioluminescence was imaged using an IVIS optical imaging system. Images were normalized and analyzed via Living Image software and signal is presented as total flux in radians (photons/s/cm^2^/steradian) over standardized regions of interest.

### Incucyte imaging and viability analysis

Tumor cells were co-cultured with indicated bacterial species at specified MOIs for 24 h as described. Co-cultures were then stained directly with HCS NuclearMask (Thermo Fisher Scientific, Waltham, MA, USA) to enable quantification of total cells, and Sytox Green (Thermo Fisher Scientific, Waltham, MA, USA) to enable quantification of dead cells. Co-cultures were then imaged using the Incucyte imaging system and dead cells were quantified as a percentage of total cells to determine viability of co-cultures. Quantification and analysis were performed using the imaging software directly which allowed bacteria to be excluded from both live and dead cell counts based on size and signal intensity.

### *De novo* bacterial sequencing and genome assembly/annotation

Single colonies of *B. thermoamylovorans* or *B. subterraneus* were cultured overnight in FAB. Bacteria were washed in PBS, pelleted, frozen, and shipped to Azenta US, Inc., for sequencing using the Short-Read Non-Human Whole-Genome Sequencing platform. DNA extraction and library preparation were performed for 2 × 150 base pairs paired-end sequencing. Samples were analyzed on the Illumina platform which produced roughly 200 million paired reads for each sample. Samples were down-sampled to 25% using the Reformat tool and duplicates were removed using the Dedupe tool, both of which are part of the BBTools package from the Joint Genome Institute (sourceforge.net/projects/bbmap/). Paired-end read libraries of approx. 20 million pairs of reads were then uploaded and further analyzed for assembly and annotation on the KBase platform (48). Adapter sequences were trimmed using Trimmomatic V0.36 (49), which was also used to filter short reads and remove low-quality bases from the first and last 10 bases of each read. Surviving paired reads were then cleaned using Prinseq v0.20.4 to remove low-complexity reads via the entropy filtering method (50). An entropy threshold was chosen so the number of surviving reads still provided >100× coverage of the total assembled genome. Cleaned reads were then assembled into contigs using Unicycler v0.4.8 for short-read bacterial genome assembly (51). Contigs were bridged using a conservative threshold to minimize the misassembly rate and contig assembly was performed to produce complete bacterial genomes. After assembly, bacterial classification was performed using the Genome Taxonomy Database-Toolkit—V2.3.2, which classifies samples using a database constructed from RefSeq and Genbank genomes (52, 53).

For annotation and enrichment analysis, the assembled genomes were then annotated using RASTtk (Rapid Annotations using Subsystems Technology toolkit) v1.073 (54–56) to call unique coding sequences (CDS/genomic feature) within the assembled genome. OrthoMCL v2.0 and Pangenome Circle Plot v1.2.0 were then used to build a pangenome and quantify the total number of CDS that were shared or unique between the two bacteria (57). For functional gene classification, protein-coding genes were identified using Prodigal and Glimmer (58, 59). These protein-coding genes were annotated using the genes present in the CoreSEED database comprised of over 1,000 diverse microbial genomes (60). Annotated genes were then further classified based on functional categories compiled on the SEED database. Each gene was annotated at four different levels with Level 4 being the identity of the coded protein and Level 1 being the broadest functional classification.

### Functional pathway enrichment comparison

CDS that were present in both genomes were identified using pangenome analysis and designated as “Shared,” while all other CDS were designated as “Unique.” One-tailed Fisher’s exact tests were used to identify SEED functional categories containing a significantly enriched number of unique CDS, where the incidence of unique CDS within a category of interest was compared with the incidence of unique CDS within the entire genome. Separate analyses were performed for each SEED hierarchal level. Categories with fewer than 10 CDS were discarded. *P* values were Benjamini-Hochberg corrected.

### Alignment and mapping of human tumor-derived bacterial sequences

For mapping of human tumor-derived bacterial sequences, patient data were obtained as part of a collaboration with Dr. Sam Aparicio and data were originally collected as part of the B-PRECISE biorepository for use in a previously published study (40). For our analyses, we used patient sequencing data that was collected under REB consent protocols (REB# H18-0113) approved for public release of raw data and deposited in the EGA archive under study ID EGAS00001006343. From this data set of TNBC patients with tumor and adjacent normal sample data, we selected all deceased patients (26 total) and then selected alive patients for the control group based on matching tumor staging to control for stage of disease progression in our downstream analyses (full patient outcome data can be made available upon request). De-identified patient sequencing data for tumor and normal samples were downloaded from the EGA archive as BAM files. Reads of human origin were discarded and all remaining unmapped reads were considered for alignment with our control or metastasis-promoting bacterial gene sets. Control and metastasis-promoting bacterial gene sets were curated by selecting all CDS from significantly enriched functional pathways (Fig. S4). The corresponding bacteria-derived DNA sequences were then compiled as either a control or metastasis-promoting gene set. Patient-derived reads were then mapped against these CDS and reads were counted using bowtie2 and featureCounts with default parameters. For reads that mapped to bacterial genes, gene ID and corresponding patient ID were determined and two-sided Fisher’s exact tests, along with calculated odds ratios, were used to compare the number of mapped reads from alive versus deceased patients against the overall outcome data of the entire patient population.

### Targeted metabolomics analysis

4T1 tumor cells were cultured with indicated bacterial species at an MOI of 1.0 for 24 h in 24-well culture plates. Co-cultures were then washed 3× in ice-cold saline solution to completely remove bacteria and any extracellular metabolites present in the media. Cellular metabolites were then extracted by adding ice-cold methanol to the cells for 20 min on ice and through 1× freeze/thaw cycle at −80°C to ensure cellular disruption. Metabolite extracts were frozen and stored on dry ice shipped for analysis by the University of Victoria Genome BC Proteomics Centre for analysis via UPLC-MRM/MS. Targeted metabolomics assays, specifically the Central Carbon Metabolism, Purine/Pyrimidine Metabolism, and Salvage Pathway, and the NAD metabolome assay were performed to quantify metabolites related to redox metabolism and energy demand. Internal standards were used to determine peak identity and to perform absolute quantitation with each analyte reported in nanomoles after normalizing to the protein concentration of each sample. For analysis of individual metabolites, metabolite levels between indicated groups were assessed using multiple *t* tests and results of *t* tests were plotted against log2 fold change values. For pathway analyses, metabolites with *P* value < 0.05 for comparisons of *B. thermoamylovoran*s to control cells were grouped into a data set that was used for pathway analysis on MetaboAnalyst. As larger differences were seen when cells exposed to *B. thermoamylovorans* were compared to cells exposed to *B. subterraneus*, metabolites with *P* value < 0.01 for comparisons of *B. thermoamylovoran*s to cells exposed to *B. subterraneus* were grouped into a data set that was used for pathway analysis on MetaboAnalyst. Full analysis details are included in the analysis reports provided in Data Set S3 (DOI: 10.5281/zenodo.11398890). Briefly, the enriched metabolite data sets were uploaded as a set of KEGG IDs, and the entire list of analyzed metabolites was uploaded as a reference library against which the enriched data sets were compared. Compounds in the enriched and reference data sets were assigned to pathways using KEGG metabolic pathways as the backend knowledgebase. Pathways that were over-represented in the enriched data sets compared to the reference data set were identified using hypergeometric statistical tests. Along with enrichment analyses, the impact the enriched metabolites have on their associated metabolic pathway was then determined using topology analysis, with impact scores calculated using relative betweenness centrality measurements.

### Statistics

The data were presented as mean ± SD or as mean ± SEM when multiple experiments were represented. Statistical analyses were conducted using Prism software (GraphPad, La Jolla, CA, USA). Statistical significance for multiple comparisons was calculated using two-way ANOVA corrected for multiple comparisons with the Holm-Sidak method unless otherwise indicated. Student’s *t* tests corrected for multiple comparisons using the Holm-Sidak method when only two data sets were being compared in a given plot. The statistical significance of survival differences on Kaplan-Meier plots was calculated using a log-rank test. Where necessary, additional statistical testing for specific analyses is described in the Materials and Methods section for a given experiment. For all tests, the significance is indicated as *P < 0.05; **P < 0.01; and ***P < 0.001.

## Data Availability

All data and resources generated in this study will be publicly available for research use. Please direct further inquiries to Dr. Shoukat Dedhar. Further information and requests for resources should be directed to and will be fulfilled by Dr. Shoukat Dedhar. This studied isolated new bacterial strains that have been saved as frozen glycerol stocks that are available upon request. Sanger sequencing data and whole bacterial genome sequencing and assembly data were deposited and published for release on the Zenodo database (DOI: 10.5281/zenodo.11398890). Patient clinical/outcome data can be made available upon request. The underlying code for this study is available on GitHub and can be accessed via the following links: https://github.com/armetcal/Metcalfe-Roach_PD_2023 and https://github.com/ZKrekhno/Breast_Cancer_Microbe.

## SUPPLEMENTAL MATERIAL

Supplemental material mbio.03595-24-s0001.pdf Supplemental figures and caption for supplemental tables.

Table S1 mbio.03595-24-s0002.txt Fold change and *P* value data for *B. thermoamylovorans*-treated cells compared to control cells.

Table S2 mbio.03595-24-s0003.txt Fold change and *P* value data for *B. subterraneus*-treated cells compared to control cells.

Table S3 mbio.03595-24-s0004.txt Fold change and *P* value data for *B. thermoamylovorans*-treated cells compared to *B. subterraneus*-treated cells.

ASM does not own the copyrights to Supplemental Material that may be linked to, or accessed through, an article. The authors have granted ASM a non-exclusive, world-wide license to publish the Supplemental Material files. Please contact the corresponding author directly for reuse.
