# Supplementary material for: Identification of intratumoral bacteria that enhance breast tumor metastasis
Source: mBio. 2025 Feb 11;16(3):e03595-24. doi: 10.1128/mbio.03595-24 (PMC11898647; doi:10.1128/mbio.03595-24)
Supplement: Supplemental material — Supplemental figures and caption for supplemental tables. [file mbio.03595-24-s0001.pdf]

## Supporting Figures and Legends for

# Bacteria from the tumor microenvironment augment metastasis in breast cancer

Zachary J. Gerbec[1,2], Antonio Serapio-Palacios[2,3,4], Avril Metcalfe-Roach[2,3,4], Zakhar Krekhno[2,3,4], Haggai Bar-Yoseph[2,3,4], Sarah E. Woodward[2,3,4], Jorge Pena-Diaz[2,3,4], Oksana Nemirovsky[1], Shannon Awrey[1], Sebastian H. Moreno[2,3,4], Sean Beatty[5,6], Esther Kong[5,6], Nina Radisavljevic[2,3,4], Mihai Cirstea[2,3,4], Shawn Chafe[1], Paul C. McDonald[1], Sam Aparicio[5,6], *B. Brett Finlay[2,3,4], and *Shoukat Dedhar[1,4,7]

*Co-corresponding authors contributed equally to this work: Shoukat Dedhar and B. Brett Finlay

**E-mail**: sdedhar@bccrc.ca, bfinlay@msl.ubc.ca

**This PDF file includes:**

Figures S1 to S4

Table Legends for Tables S1 to S3

## Supporting Figures and Figure Legends

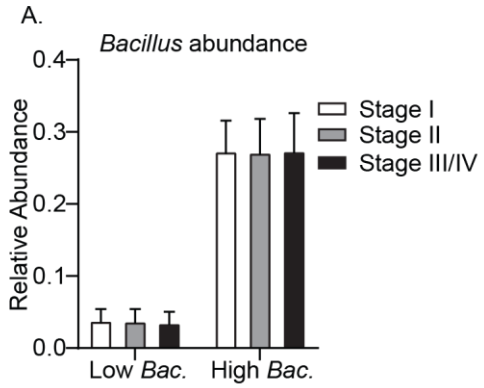


*Figure S1 Gerbec et al.,*

**Figure S1. Relative abundance of *Bacillus* across disease stages.**

(A) For the low and high quartiles of patients when separated based on *Bacillus* expression, relative abundance of *Bacillus* within the specified disease stage was measured to determine if abundance varied across disease stages within the patient populations for which outcome data were compared.

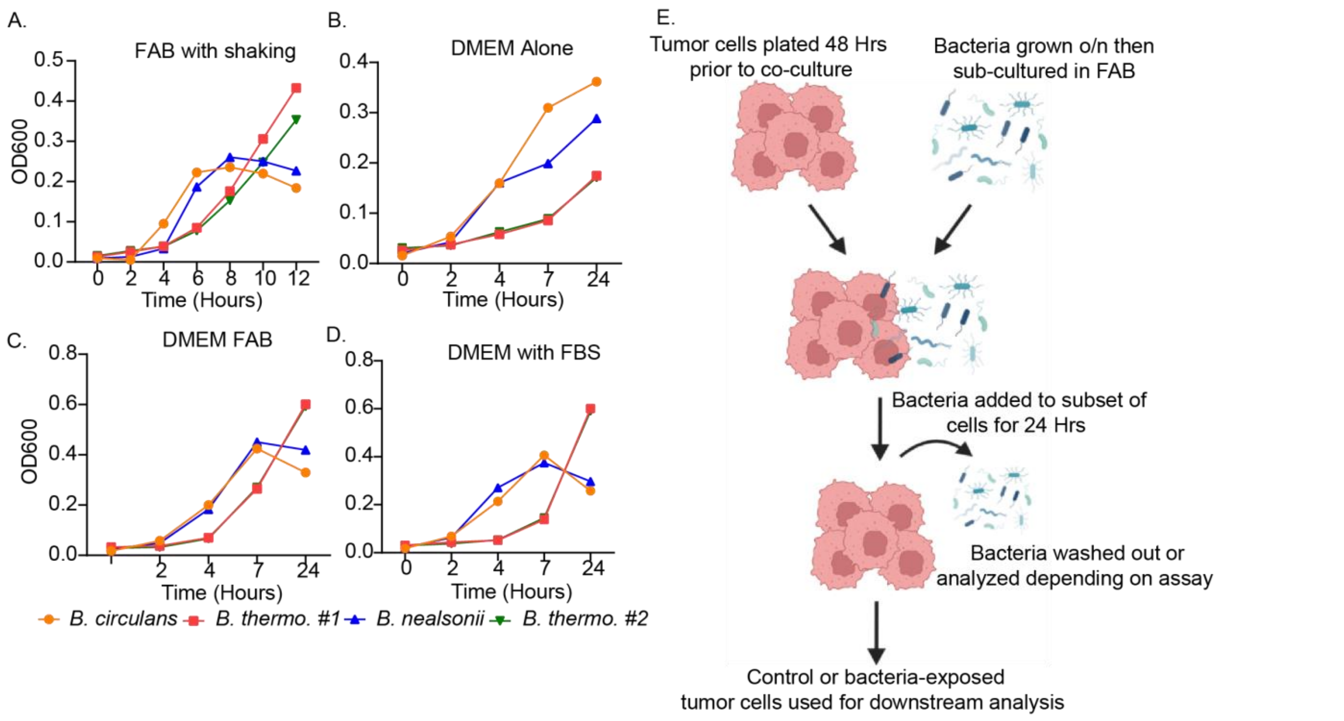


**Figure S2. Bacteria-4T1 culture model development.**

(A-D) Growth of various Bacillus species isolated from 4T1 tumors was evaluated via OD600 measurement in different growth medias over indicated time frames to determine consistency between culture conditions. Normal bacterial culture conditions (A) were used as controls with DMEM alone and DMEM with FAB (B,C) representing intermediates between bacterial and mammalian conditions and finally DMEM with FBS (D) representing standard tumor cell culture conditions. (E) A schematic depicting method for bacteria/tumor cell co-culture is shown as a general outline for the co-culture methodologies with slight adaptations made depending on each experiment.

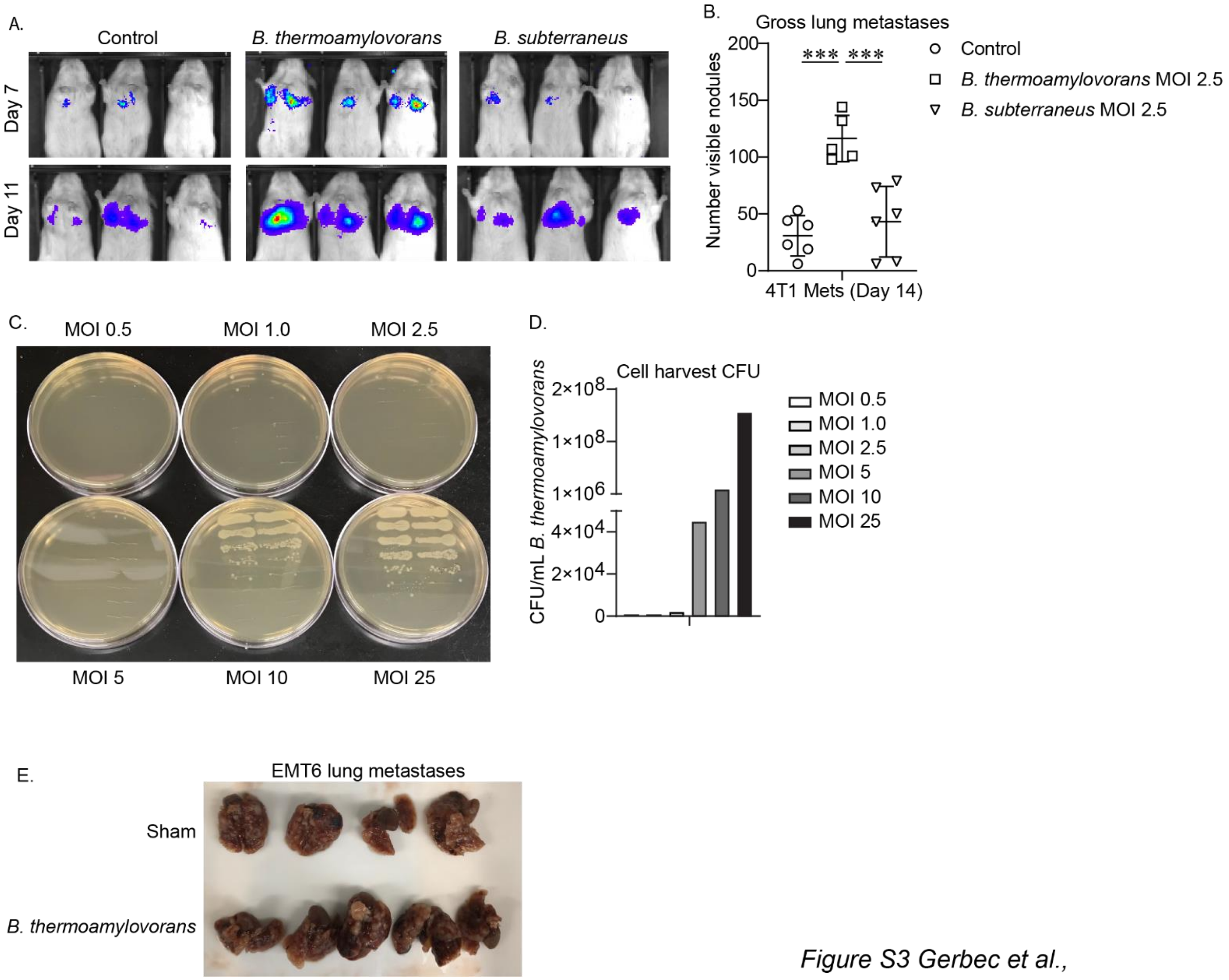


**Figure S3. 24-Hour *B. thermoamylovorans* exposure augments 4T1 metastatic potential.**

(A,B) Luc+ 4T1 tumor cells were cultured with vehicle (control) or with indicated bacterial species at an MOI of 2.5 for 24 hours. Bacteria were then washed away and cells were harvested and injected through the lateral tail vein into NSG mice. After two weeks whole lung tissues were removed and visible metastatic nodules were counted to directly quantify metastatic disease (n=5-6 per group). (C,D) 4T1 tumor cells were co-cultured with *B. thermoamylovorans* at indicated MOIs for 24 hours. Bacteria/tumor cell co-cultures were then rinsed, trypsinized, and washed 2x in PBS using the same procedure used for intravenous inoculation. Suspensions were then plated on fastidious anaerobe agar plates and bacterial CFU was quantified to determine if bacteria were injected along with cellular inoculum. (E) EMT6 tumor-bearing mice were inoculated intratumorally with *B. thermoamylovorans* as described in **Figure 3**. Animals were euthanized 2 days after the final bacterial injection and

lungs were resected and imaged as shown. Data are presented as mean +/- SEM. ***, $p < 0.001$.

A.

| Pathways enriched based on unique CDS | | | | | |
|---|---|---|---|---|---|
| | SEED Category (ontology level) | p-value | p-adjusted | Unique Genes | Total |
| *B. thermoamylovorans* | Di- and oligosaccharides (2) | 2.62E-05 | 0.0014 | 23 | 42 |
| | DNA Metabolism (2) | 0.00011 | 0.0028 | 11 | 15 |
| | Monosaccharides (2) | 0.00016 | 0.0028 | 22 | 43 |
| | Membrane Transport (2) | 0.00060 | 0.0079 | 18 | 35 |
| | Cell wall of Mycobacteria (2) | 0.00075 | 0.0080 | 10 | 15 |
| | Nitrogen Metabolism (2) | 0.0016 | 0.014 | 12 | 21 |
| | Capsular and extracellular polysaccharides (2) | 0.0022 | 0.016 | 11 | 19 |
| | Catabolism of aromatic compounds 2) | 0.020 | 0.13 | 10 | 21 |
| | Sugar Alcohols (2) | 0.040 | 0.24 | 10 | 23 |
| | Restriction Modification System (3) | 0.00012 | 0.0037 | 10 | 13 |
| | Linker Unit-arabinogalactan synthesis (3) | 0.00012 | 0.0037 | 9 | 11 |
| | Rhamnose containing glycans (3) | 0.0004 | 0.0082 | 8 | 10 |
| | Coenzyme B12 biosynthesis (3) | 0.020 | 0.30 | 6 | 10 |
| | Beta-glucoside metabolism (3) | 0.034 | 0.35 | 6 | 11 |
| | Pyruvate-Alanine-Serine interconversion (3) | 0.034 | 0.35 | 6 | 11 |
| *B. subterraneus* | Electron donating reactions (2) | 7.56E-10 | 4.24E-8 | 25 | 29 |
| | Stress Response (2) | 1.24E-7 | 3.48E-6 | 26 | 35 |
| | Respiration (2) | 0.00013 | 0.0024 | 18 | 27 |
| | Metabolism of central aromatic intermediates(2) | 0.0053 | 0.075 | 8 | 11 |
| | Phosphorus Metabolism (2) | 0.0091 | 0.10 | 10 | 16 |
| | ABC Transporters (2) | 0.012 | 0.011 | 8 | 12 |
| | NAD and NADP (2) | 0.033 | 0.27 | 11 | 21 |
| | Nitrogen Metabolism (2) | 0.038 | 0.27 | 8 | 14 |
| | Antibiotics and toxins resistance (2) | 0.047 | 0.27 | 16 | 35 |
| | Catabolism of aromatic compounds (2) | 0.048 | 0.27 | 11 | 22 |
| | SigmaB stress response regulation(3) | 7.33E-7 | 4.32E-5 | 12 | 12 |
| | Respiratory Complex 1 (3) | 2.46E-6 | 7.09E-5 | 11 | 11 |
| | Formate hydrogenase (3) | 7.86E-6 | 0.00016 | 10 | 10 |
| | Bacterial hemoglobins (3) | 3.45E-5 | 0.00051 | 12 | 14 |
| | NAD and NADP cofactor biosynthesis (3) | 0.040 | 0.45 | 10 | 19 |
| | Biogenesis of c-type cytochromes (3) | 0.046 | 0.45 | 7 | 12 |

Pathway enrichment results based on the number of unique genes found in a given SEED functional classification are shown. Classifications are designated as 1 of 4 ontology levels, with 1 being the most broad level and 4 being a specific gene. Classification levels 2 and 3 are shown here along with the number of total and unique genes in a given category. Enrichment was calcualted using Fisher's Exact tests and p values are shown before and after correction for multiple comparisons to provide a comprehensive view of the genetic differences between the two bacteria.

*Figure S4 Gerbec et al.,*

**Figure S4. Metastasis-promoting *B. thermoamylovorans* harbors unique functional genes.**

De novo sequencing and genome assembly was performed on *B. thermoamylovorans* and *B. subterraneus* as described in the materials and methods section. Bacterial coding sequences (CDS) identified were mapped onto functional genes and compiled into categories based on the SEED database for bacterial genomic analysis. Unique and shared coding sequences were identified and quantified for each species. After SEED gene and category-level classification, Fisher's exact test was used to identify pathways that were significantly over-represented in *B. thermoamylovorans* or *B. subterraneus* based on the number of unique

CDS present in a given pathway compared to the number of unique CDS present in the entire genome. Table shows all functional pathways with p value above 0.05 prior to FDR correction with full results available in **Supporting Datasets S7** (DOI 10.5281/zenodo.11398890).

## Supplemental Table Legends

**Figure S1-3. Fold-change and P value data for individual metabolite comparisons. Tables S1-S3** were used to generate data in **Figure 4**. Tables show levels of individual metabolites in nanomoles after normalization to sample protein concentration and log2 transformation along with the statistical significance of the difference in metabolite levels between indicated groups when assessed using multiple t tests on the individual metabolites. **Table 1** contains data for *B. thermoamylovorans*-treated cells compared to control cells. **Table 2** contains data for *B. subterraneus*-treated cells compared to control cells. **Table 3** contains data for *B. thermoamylovorans*-treated cells compared to *B. subterraneus*-treated cells.
